# Ultraviolet Radiation in the Remediation of Cr(VI)-Contaminated Soil by Corn Stover

**DOI:** 10.3390/toxics14090754

**Published:** 2026-08-26

**Authors:** Yiping Guo, Shihang Ni, Qianqian Zhang, Weigao Zhao, Peng Liu, Yunchao Dai, Hui Wang

**Affiliations:** 1 School of Ecology and Environment, North China University of Water Resources and Electric Power, Zhengzhou 450046, China; 2 Henan Engineering Research Center of Water Pollution and Soil Damage Remediation, Zhengzhou 450046, China; 3 Department of Environmental Engineering, School of Environmental Science and Engineering, Tianjin University, Tianjin 300072, China; 4 College of Natural Resources and Environment, Northwest A&F University, Yangling 712100, China; 5 School of Chemistry & Chemical Engineering, Henan University of Science and Technology, Luoyang 471023, China

**Keywords:** ultraviolet radiation, Cr(VI), soil remediation, corn stover, mechanism

## Abstract

Ultraviolet (UV) radiation was selected in this study to evaluate its assistant remediation effects on Cr-contaminated soil. Various remediation materials, including corn stover (CS), corn stover biochar (CSB), polypyrrole-modified 44 corn stover (PPy-CS), and polypyrrole-modified corn stover biochar (PPy-CSB), were employed to evaluate their synergistic effects with UV radiation. The results showed that UV radiation increased the toxicity characteristic leaching procedure (TCLP) Cr(VI) when no other remediation was added to the contaminated soil. However, when the remediation materials were added, with UV radiation, the contents of TCLP-Cr(VI) decreased, while the contents of Cr(III) increased, and the removal rates of TCLP-Cr(VI) in contaminated soil treated by CSB, PPy-CSB, CS, and PPy-CS were 18.7%, 7.6%, 11.0%, and 8.2% higher than those of the non-irradiated groups, respectively. Notably, the CS group demonstrated superior efficacy in Cr(VI) removal compared with CSB under UV irradiation. Meanwhile, characteristic analysis including electron paramagnetic resonance (EPR), Fourier-transform infrared spectroscopy(FTIR) and X-ray photoelectron spectroscopy(XPS) assisted us in finding reaction mechanisms, which implied that UV irradiation could enhance the content of oxygen free radicals on the material surface and effectively activate the reactions between Cr(VI) and remediation materials. This study verified that UV irradiation could also be a promising tool in the remediation of heavy-metal-contaminated soil.

## 1. Introduction

Chromium pollution is a globally recognized environmental concern. It is released into soil and natural water through wastes discharged from various sources, including electronics, electroplating, metallurgical sources, leather tanning, and pharmaceutical industries [1,2,3]. The discharged chromium is mainly divided into Cr(VI) and Cr(III). Notably, the toxicity of Cr(VI) is nearly 500 times higher than that of Cr(III) [4,5], and at the same time, Cr(VI) has strong carcinogenic and mutagenic properties [6,7]. Because of the unstable valence state of chromium, the remediation process often results in incomplete repair and undesirable morphological transformations, which increase the difficulty of the treatment. Meanwhile, most current research is focused on the remediation of water-soluble hexavalent chromium, whereas research on the remediation of hexavalent chromium in soil is lacking [8,9].

Biochar is a carbon-rich material produced from biomass and is considered an excellent remediation agent due to its large specific surface area, strong adsorption capacity, and stable structure, offering significant advantages over raw biomass [10,11]. To further enhance its performance, various modifications have been developed, including UV radiation modification, acid–base modification, supporting metals and their oxides, and organic modification [12,13,14]. Among these, UV radiation modification stands out as a particularly competitive and clean approach. It can not only enhance the adsorption capacity of biochar for pollutants by increasing oxygen-containing functional groups such as -OH, -COO, -CO, and -COOH but also avoid secondary pollution during the modification process [15,16,17]. For instance, Li et al. found that coconut shell biochar irradiated with UV light at 365 nm for 16 h had the best adsorption effect on Cd(II) in soil [18]. Similarly, Wang et al. observed that the adsorption of Pb(II) and Cd(II) in solution by biochar after UV irradiation was 136.0% and 25.3% higher than that before UV irradiation [13]. Nevertheless, despite these promising results, the application of UV-modified biochar is predominantly confined to wastewater treatment. Its potential for remediating the more complex and challenging case of Cr(VI) in soil is severely underexplored.

Our previous research demonstrated that polypyrrole-modified corn stover (PPy-CS) and polypyrrole-modified corn stover biochar (PPy-CSB) exhibited excellent remediation performance for Cr(VI) in soil [19]. Building upon this foundation, as well as seeking to address the persistent challenge of Cr(VI) contamination, the new synergistic strategy of UV radiation is proposed. Its performance is examined here, and characteristic analyses such as energy dispersive X-ray photoelectron spectroscopy (XPS) analyses, Fourier-transform infrared spectroscopy (FTIR) and electron paramagnetic resonance (EPR) were carried out. This study can enrich the application of UV radiation in the area of environmental pollution treatment.

## 2. Materials and Methods

### 2.1. Materials

Soil samples used in this study were collected from the top layer (0–20 cm) of the campus experimental field (113°48′ E, 34°46′ N) in Zhengzhou, Henan, China, and the soil type was meadow-cinnamon soil. Corn straw was sourced from Zhengzhou, Henan Province, China. The basic physical and chemical properties of the soil are shown in Table 1, where the indicators of organic matter, soil bulk density, and alkaline nitrogen were tested according to the methods in soil testing standard (NY/T 1121-2006) [20] issued by the Agricultural Industry Standards of the People’s Republic of China, and total chromium, total lead, and total copper were analyzed following the soil and sediment-determination of copper, zinc, lead, nickel and chromium-Flame atomic absorption spectrophotometry (HJ 491-2019) [21] issued by the National Environmental Protection Standards of the People’s Republic of China using an atomic absorption spectrophotometer (TAS-990 SUP, Persee, Beijing, China).

### 2.2. Methods

#### 2.2.1. Preparation of Remediation Materials

The corn stover biochar was produced by heating dried corn stover sieved to 425 µm in a muffle furnace at a relatively low and environmentally friendly temperature of 350 °C. Meanwhile, polypyrrole-modified corn stover biochar (PPy-CSB) was also used, and this material was prepared using corn stover biochar, pyrrole, a hydrochloric acid solution, and ammonium persulfate. It exhibited significantly better performance in the remediation of Cr(VI)-contaminated soil in our previous studies [19]. In addition, corn stover biochar (CSB) was replaced with corn stover (CS), and polypyrrole-modified corn stover (PPy-CS) was prepared following the method used for PPy-CSB preparation.

#### 2.2.2. Experiments

According to the Soil Environmental Quality-Risk Control Standard for Soil Contamination of Agricultural Land of China (GB 15618–2018) [22], when the pH of farmland soil exceeds 7.5, the risk screening value for hexavalent chromium is set at 350 mg·kg^−1^, while the risk control value is set at 1300 mg·kg^−1^. In this study, an initial soil pollution concentration of 600 mg·kg^−1^ was selected. This concentration was above the screening value, belonging to moderate to severe pollution scenarios, yet below the risk control value. Firstly, the soil samples were pretreated by adding analytical-grade K_2_CrO_4_ reagent to achieve the calculated Cr(VI) concentration in the soil of 600 mg·kg^−1^. Then, the contaminated soil was kept at room temperature for one week with humidity at 60%~70% of the field water-holding capacity.

In the experiments, the above soil was firstly mixed with 5% CS, CSB, PPy-CS, and PPy-CSB, respectively. At the same time, no additions were added to the soil in the control group. After being stirred evenly, the mixtures were kept at 60~70% of the field water-holding capacity, where the field water-holding capacity of soil was determined via the cutting ring method specified in the Agricultural Trade Standard of China (NY/T 1121.22-2010) [23]. Then, the mixed soil samples were spread evenly to a thickness of approximately 2 mm and placed 30 cm directly below a UV lamp with a power of 30 W and a wavelength of 254 nm for irradiation for 0 min, 10 min, 20 min, 30 min, 60 min, and 120 min, respectively [24,25,26]. UV irradiation was performed using a low-pressure mercury lamp with a rated electrical power of 30 W, whose principal emission wavelength was 254 nm. Since 254 nm is the dominant characteristic emission line of the low-pressure mercury lamp, no wavelength filters or monochromator was required. The irradiance at the sample surface (30 cm below the lamp) at 254 nm was approximately 0.5 mW/cm^2^. A wavelength of 254 nm was chosen referencing the published research, which has reported that it could effectively excite the electronic transition and promote the reduction of Cr(VI) to Cr(III) [27]. Samples were taken at various time intervals, air-dried naturally and passed through a 2 mm sieve for testing. The experiments were divided into two groups of irradiation and non-irradiation. Each condition was conducted in triplicate, and average values were used for data analysis.

#### 2.2.3. Characterization Methods

The samples collected for characterization were irradiated by a UV lamp for 2 h, and non-irradiated materials served as controls. The changes of free radicals in the soil after ultraviolet radiation were detected by electron paramagnetic resonance spectroscopy (EPR) (Bruker EMXplus-6/1, Bruker BioSpin GmbH, Rheinstetten, Germany). The functional groups on the remediation materials were identified using Fourier-transform infrared spectroscopy (FTIR, Nicolet iS5, Thermo Fisher, Waltham, MA, USA). X-ray photoelectron spectroscopy (XPS, Thermo Fisher Scientific ESCALAB 250Xi, Waltham, MA, USA) was used to characterize the types and valence states of the elements in the soil.

#### 2.2.4. Data Analysis

The content of Cr(VI) was determined by the method specified in the National Environmental Protection Standards of the People’s Republic of China (HJ 1082-2019) [28] for soil and sediment determination of Cr(VI) using alkaline digestion and flame atomic absorption spectrometry. The toxicity characteristic leaching procedure (TCLP) method was adopted to test the availability of Cr(VI). This determination was performed using the standard method of the Environmental Protection Agency (EPA) in the USA (US Environmental, 1992), which is a widely used method for evaluating the ecological risk of heavy metals in soil and has the characteristics of simplicity and rapidity [29,30]. The specific detection steps were as follows. Firstly, since the initial pH of the tested soil exceeded 5.0, TCLP extraction fluid with a pH of 2.88 ± 0.05 was selected in accordance with the EPA method. The extractant was prepared by dissolving glacial acetic acid in deionized water, and the pH was adjusted using HNO_3_ (1 mol·L^−1^) or NaOH (1 mol·L^−1^) to maintain the solution pH within the range of 2.88 ± 0.05. The soil samples were mixed with the extractant solution in a ratio of 1:20, and then the mixtures were shaken at 150 r·min^−1^ for 18 h at room temperature; after that, they were centrifuged and filtered through a 0.45 μm filter membrane for detection.

The adsorption capacity of remediation materials was calculated by the following formula:

(1) qt=C0−Ct×Vm

where $q_{t}$ (mg·g^−1^) is the adsorption capacity at time *t*, $C_{0}$ (mg·L^−1^) is the concentration of Cr(VI) in the TCLP extractions before treatment, and $C_{t}$ (mg·L^−1^) is the concentration of Cr(VI) in the TCLP extractions at time *t* after treatment. The term ‘adsorption capacity’ ($q_{t}$) used in this study refers to the apparent amount of Cr(VI) removed from the TCLP leachate per unit mass of the amendment material, reflecting the combined effects of surface adsorption, immobilization, and chemical reduction of Cr(VI) to Cr(III).

The Cr content in the soil was determined by flame atomic absorption spectrophotometry, and the TCLP-Cr(VI) content was determined by a diphenylcarbonyl dihydrazide spectrophotometer after the extraction using the TCLP method.

The stabilization efficiency of Cr(VI) was calculated using the following formula:

(2) η=ω0−ωeω0×100%

where η is the stabilization efficiency (%), $\omega_{0}$ is the concentration of Cr(VI) in the TCLP extract before remediation (mg·kg^−1^), and $\omega_{e}$ is the concentration of Cr(VI) (mg·kg^−1^) in the TCLP extract after remediation.

The experimental data were analyzed using Excel 2019 and Avantage 5.9931. Figures were plotted using Origin 2018.

## 3. Results and Discussion

### 3.1. Effects of Ultraviolet Radiation on TCLP-Cr(VI) Under Different Remediation Materials in the Soil

The properties of the simulated contaminated soil were tested and are presented in Table 2. It can be observed that although the initial set concentration of Cr(VI) was 600 mg/kg, the actual Cr(VI) concentration tested in the soil was only around 582.36 mg·kg^−1^ immediately, and even after one week, the total Cr(VI) concentration was only around 512.73 mg·kg^−1^. The decreasing content of Cr(VI) may have resulted from reduction reactions by some reducing substances such as Fe^2+^, Mn^2+^, and S^2−^ that occur in natural soil. At the same time, the average Cr(VI) content in the TCLP extractions was also much lower, implying that part of the Cr(VI) was fixed firmly in the natural soil. It was also found that the soil pH value increased from 7.80 to 8.13 compared with the background soil, which was reasonable because the K_2_CrO_4_ added in the soil was alkaline [29].

**Figure 1:**
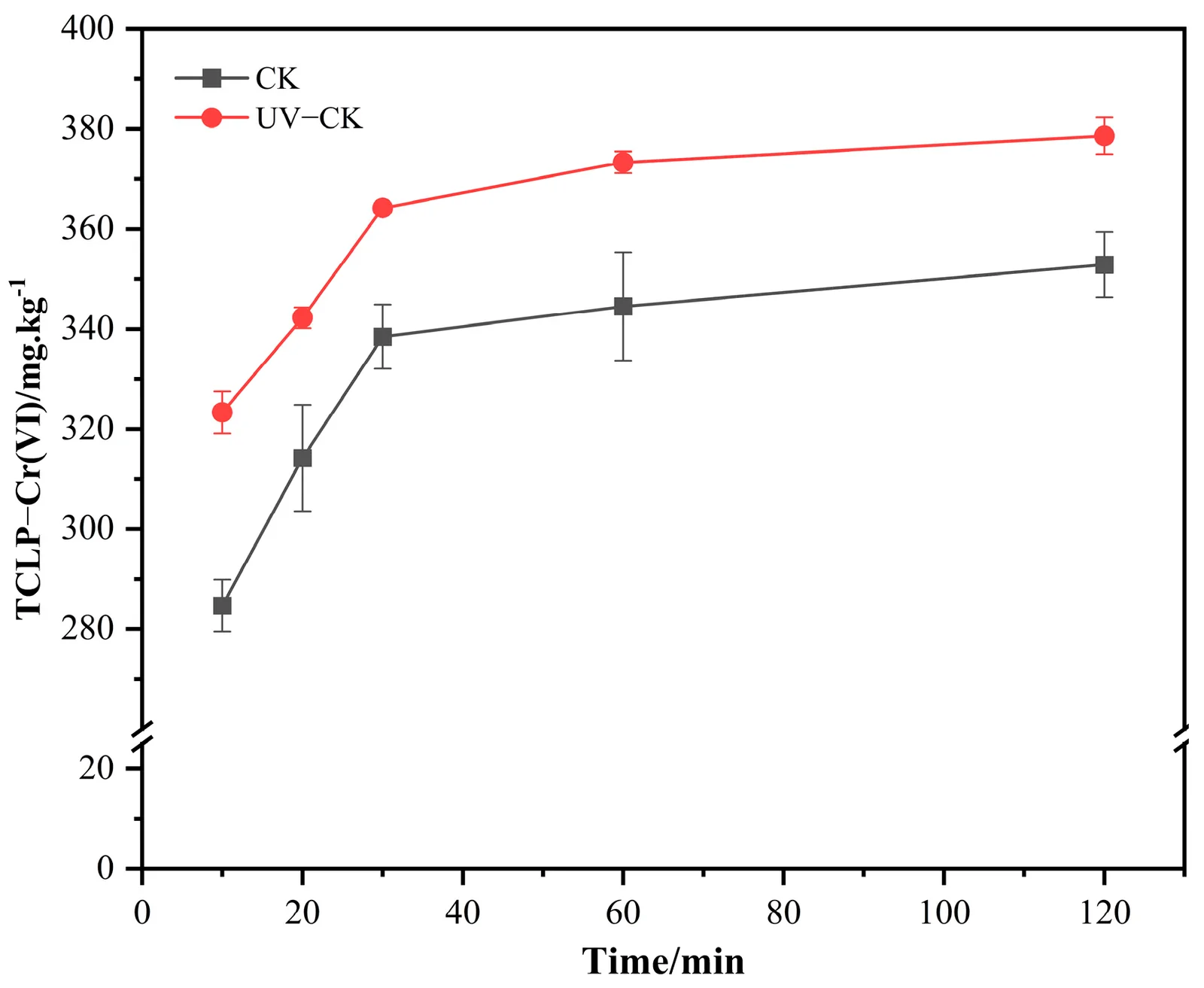
TCLP-Cr (VI) changes with and without UV irradiation under no remediation materials.

In the experiments, the above soil samples were mixed with different remediation materials such as CS, CSB, PPy-CS and PPy-CSB. The materials of PPy-CS and PPy-CSB were employed because they contained conjugated long chain structures, as well as advantageous properties such as ion exchange and redox properties, and their good performance has been demonstrated in the remediation of soil Cr(VI) in our former study [19]. All the soil samples with remediation materials were divided into irradiation groups and non-irradiation groups. Firstly, Figure 1 shows that TCLP-Cr(VI) changes with and without UV irradiation under no remediation materials in the soil samples. It can be seen that the TCLP-Cr(VI) values increased by around 20–40 mg·kg^−1^ under UV irradiation compared to the non-irradiated control, indicating that UV irradiation induced the oxidation of Cr(III) to Cr(VI) in the absence of remediation materials.

**Figure 2:**
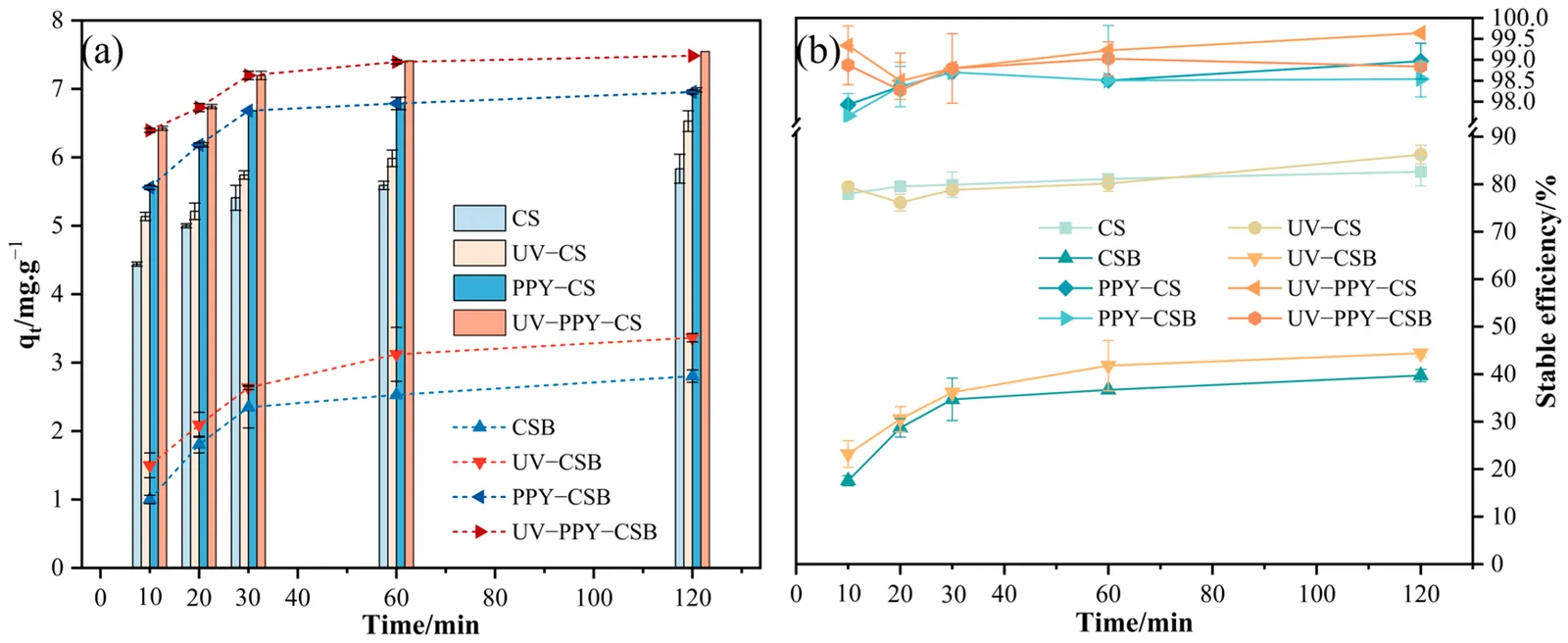
Adsorption capacity and stabilization efficiency of remediation materials for Cr(VI) in contaminated soil with and without UV irradiation: (**a**) adsorption capacity, (**b**) stabilization efficiency.

The impact of various treatment environments on Cr (VI) in soil, both prior to and following UV irradiation, varies with reaction time, as illustrated in Figure 2. It is obvious that the adsorption capacity had higher values with UV irradiation than those without UV irradiation under all the remediation conditions. Moreover, it can be seen that the treatment effects under the remediation with CS were better than those under CSB, while the treatment effects were similar under both the PPy-CS and PPy-CSB. At the same time, the best effects occurred under both UV-PPy-CS and UV-PPy-CSB. All the adsorption capacities increased remarkably in the initial 30 min under all the remediation conditions; after that, the increasing trends of the values became more gentle and gradually reached equilibrium around 120 min. Finally, the adsorption capacities were around 2.83, 3.36, 6.95, 7.48, 5.58, 6.19, 6.98, and 7.55 mg·g^−1^ under remediation conditions of CSB, UV-CSB, PPy-CSB, UV-PPy-CSB, CS, UV-CS, PPy-CS, and UV-PPy-CS, respectively. It is clear that the adsorption capacities with UV irradiation increased by 18.7%, 7.6%, 11%, and 8.2% under remediation conditions of CSB, PPy-CSB, CS, and PPy-CS, respectively, compared with those without UV treatment. It can be concluded that the UV irradiation could activate the reactions between Cr(VI) and remediation agents in the soil, and the UV-CS achieved a Cr(VI) removal performance comparable to that of the PPy CSB. The stabilization efficiency of Cr(VI) exhibited the most pronounced increases under CSB and UV-CSB treatments, reaching 22.15% and 21.22% at equilibrium compared to the initial values (0 min), respectively. However, it should be noted that the adsorption kinetics of CSB and UV-CSB were relatively slower than those of other materials, as evidenced by the gradual increase in adsorption capacity over time (Figure 2a). The stabilization efficiency of CS is higher than that of CSB. Meanwhile, the stabilization efficiency of both materials improved after UV irradiation.

### 3.2. XPS Analysis

**Figure 3:**
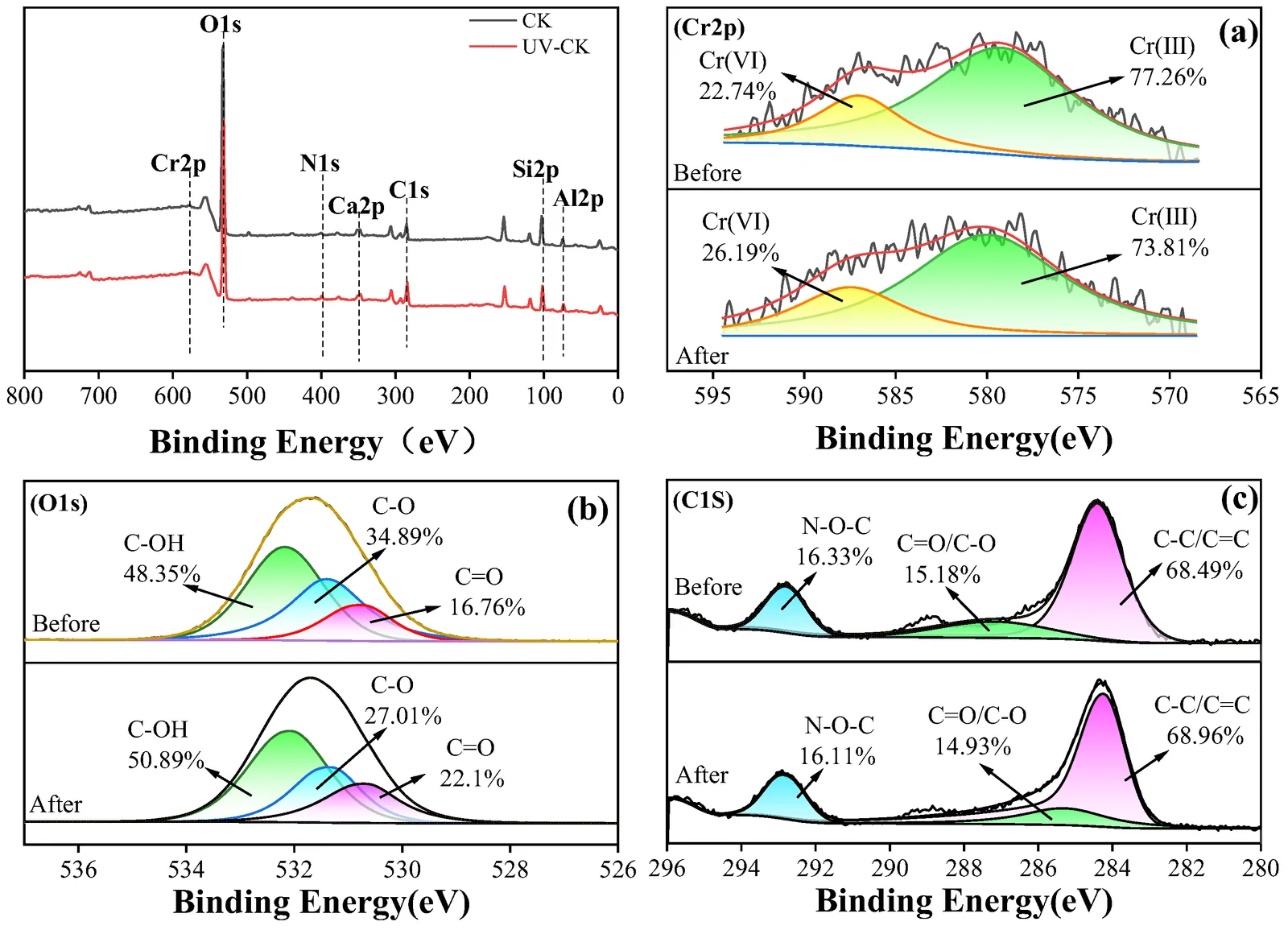
XPS of contaminated soil before and after the UV treatment under no remediation. (**a**) Cr2p spectrum; (**b**) O1s spectrum; (**c**) C1s spectrum.

XPS analysis was employed to assist in figuring out the reaction mechanisms in the remediation process. As shown in Figure 3, the survey spectra of the contaminated soil before and after UV irradiation (without any remediation) revealed the presence of C, N, O, Ca, Si, Al, and Cr, while the total elements and peak positions showed no significant changes before and after UV irradiation. However, according to the peak-differentiation-imitating analysis of element Cr, it was found that the electronic peaks of Cr(VI) and Cr(III) corresponded to 587.0 ev and 579.2 ev, and the contents of Cr(VI) and Cr(III) before UV irradiation were 22.74% and 77.26%, respectively, while the values were 26.19% and 73.81% after UV irradiation. The increasing content of Cr(VI) and decreasing content of Cr(III) indicated that UV irradiation could promote some Cr(III) into Cr(VI) in the contaminated soil under the situation of no remediation. Concurrently, the analysis of element O revealed that the C=O and C-OH groups increased from 16.76% and 43.35% to 22.10% and 50.89%, respectively, while the C-O group decreased from 34.89% to 27.01%. The C-O component in the O1s spectra mainly refers to the ether bond (C-O-C). Other functional groups, including C-C/C=C, N-O-C, and C=O/C-O, showed only minor changes. It could be inferred that under UV irradiation, the oxygen in the air participates in photochemical reactions and generates reactive oxygen species, which simultaneously oxidize Cr(III) to Cr(VI) and the organic matter in soil. The decrease in C-O-C (ether bonds) and the increase in C-OH and C=O groups reflect the photo-oxidative degradation of soil organic matter, which occurs in parallel with the oxidation of Cr(III).

**Figure 4:**
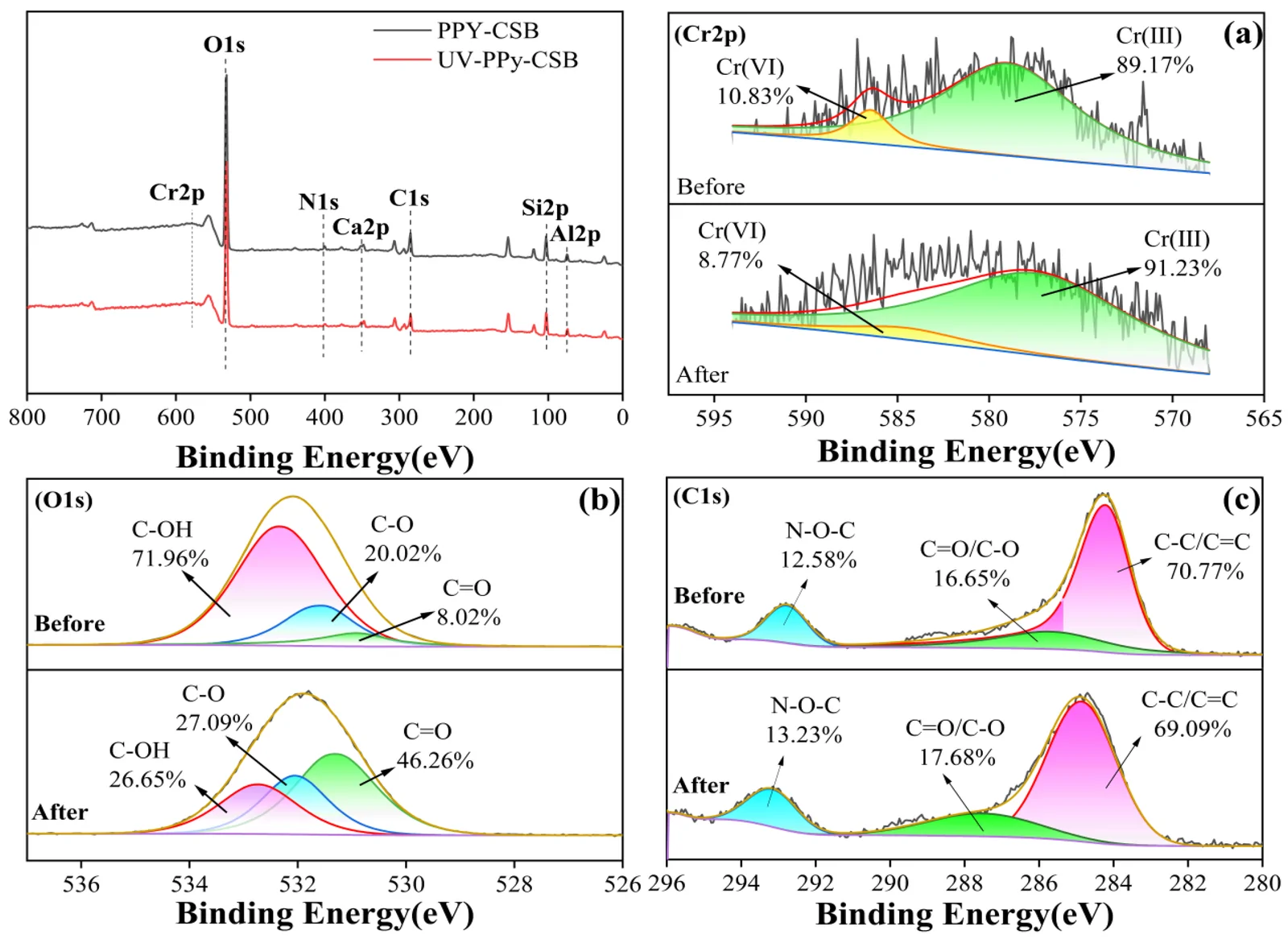
XPS spectra of contaminated soil before and after UV irradiation under PPy-CSB remediation. (**a**) Cr2p spectrum; (**b**) O1s spectrum; (**c**) C1s spectrum.

Concurrently, Figure 4 depicts the XPS of contaminated soil with and without UV irradiation under PPy-CSB remediation. It can be seen that the UV irradiation resulted in a slight decrease in Cr(VI) content from 10.83% to 8.77%, while Cr(III) increased from 89.17% to 91.23%. Particular attention should be paid to the content of the C-OH group, which showed a significant decrease from 72.96% to 26.65%; meanwhile, the C-O and C=O groups increased from 20.20% and 8.02% to 27.09% and 46.26%, respectively. It could be inferred that the reduction of Cr(VI) to Cr(III) may be coupled with the oxidation of carbon-containing groups on the material surface, which is mediated by oxygen in the air under UV irradiation.

**Figure 5:**
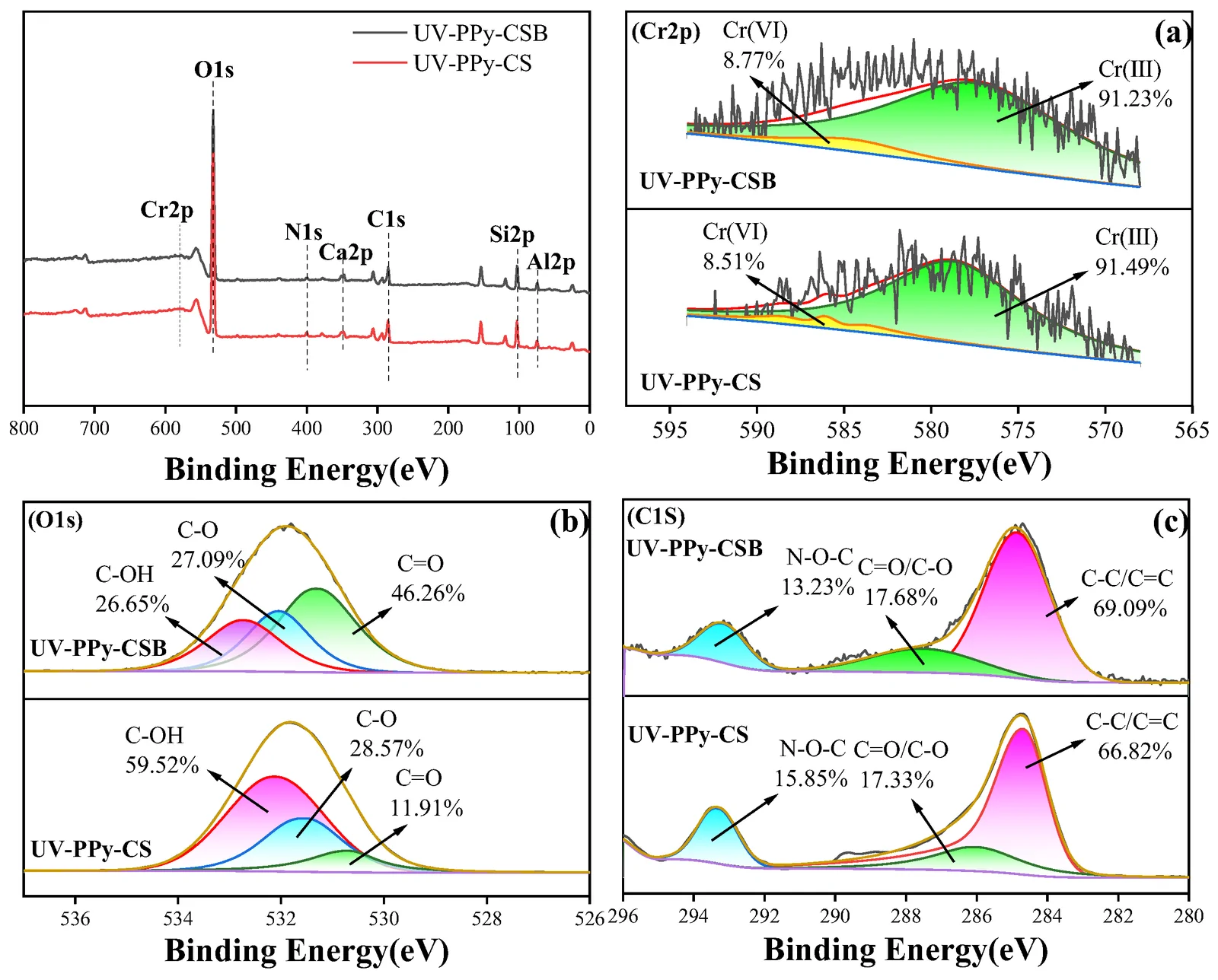
XPS spectra of contaminated soil with UV irradiation under the PPy-CSB and PPy-CS. (**a**) Cr2p spectrum; (**b**) O1s spectrum; (**c**) C1s spectrum.

The XPS analysis in Figure 5 reveals that the Cr and C contents were similar in the contaminated soil treated with UV-PPy-CSB and UV-PPy-CS. In contrast, the O1s spectra revealed distinct distributions of oxygen functional groups: the C-OH, C-O, and C=O groups were 26.65%, 27.09%, and 46.26%, respectively, under UV-PPy-CSB and 59.52%, 28.57%, and 11.91%, respectively, under UV-PPy-CS. The most pronounced differences occurred in the C-OH and C=O groups, indicating distinct oxygen reaction pathways for UV-PPy-CSB and UV-PPy-CS. The substantially lower C=O content in UV-PPy-CS may be attributed to the generation of free radicals under UV irradiation, which facilitates chain transfer and redox reactions with adjacent molecules [31].

### 3.3. Electron Paramagnetic Resonance Analysis

**Figure 6:**
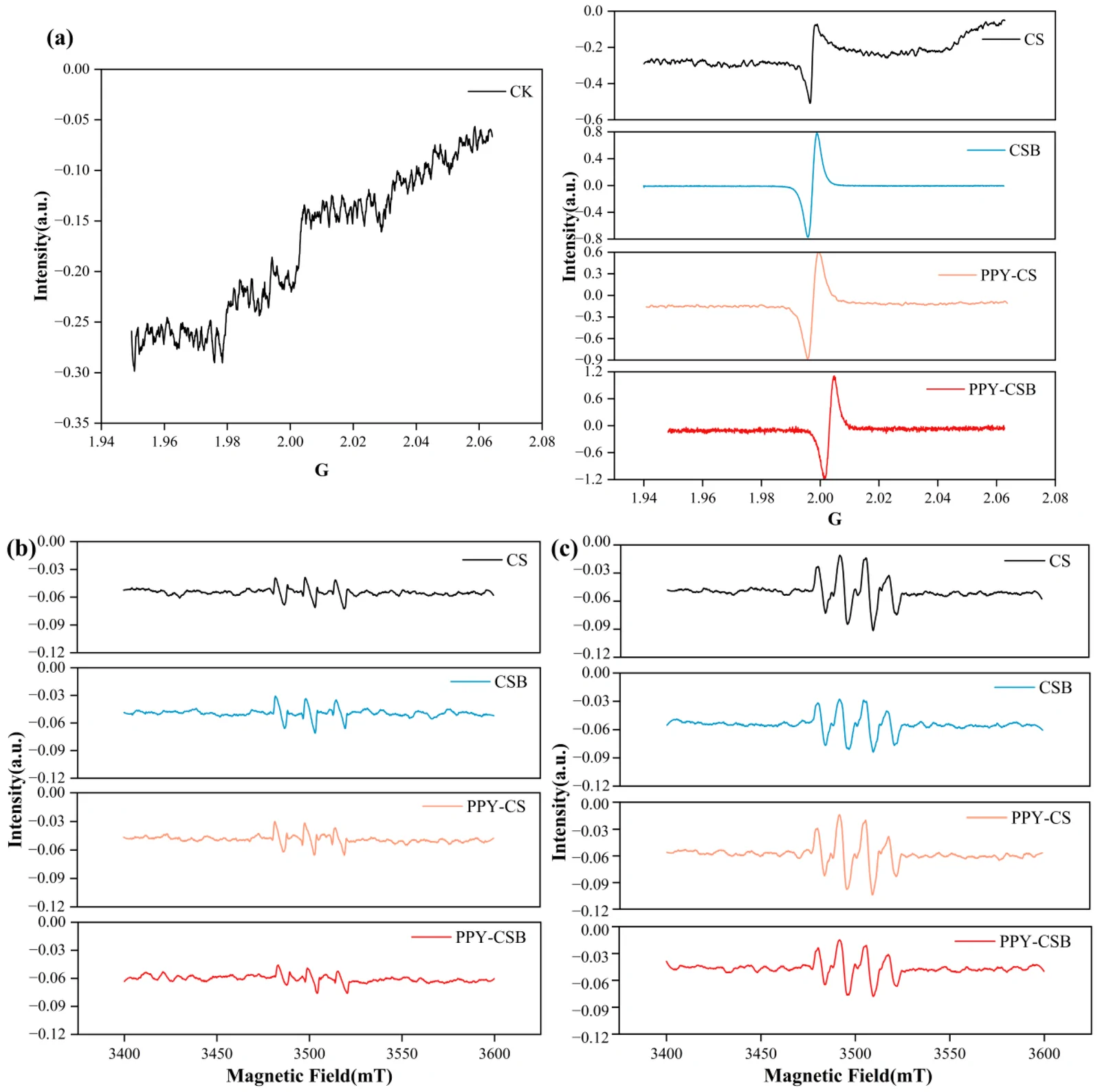
EPR spectra of contaminated soil under remediation materials under UV irradiation. (**a**) Intrinsic persistent free radicals, (**b**) EPR spectra of spin-trapped ^1^O_2_ (TEMP-TEMPO adduct), (**c**) EPR spectra of spin-trapped O_2_^−^· (DMPO-OOH adduct).

Electron paramagnetic resonance (EPR) spectroscopy, coupled with the spin-trapping technique, was employed to analyze the UV-irradiated remediated soil by detecting the spectra of stable nitroxide radical adducts formed from the reaction between spin traps and reactive oxygen species. The EPR spectra of persistent free radicals (PFRs⋅) in the CK (blank soil), CS, CSB, PPy-CS and PPy-CSB samples are shown in Figure 6a. All remediation materials exhibited a typical signal of carbon-centered persistent free radicals at g ≈ 2.003 [32]. This signal was generated by the excitation of conjugated structures on the material surfaces under ultraviolet irradiation and acted as the core electron supplier for the reduction of Cr(VI). The CK showed almost no obvious EPR signal, only baseline noise, indicating that the concentration of intrinsic free radicals in natural soil was extremely low and could be ignored. The CS presented a relatively low intensity of UV-induced persistent free radicals due to the lack of continuous conjugated photosensitive structures. The CSB possessed a conjugated carbon skeleton owing to the promoted aromatic ring condensation during high-temperature pyrolysis [33]. It showed a fast recombination rate of surface photogenerated electrons, leading to a free-radical signal only slightly higher than that of CS. After modification with PPy, the free-radical signals of both PPy-CS and PPy-CSB were greatly enhanced, while the signal intensities of these two modified materials showed no significant difference.

Figure 6b,c show the EPR spectra of the spin-trapped adducts for ^1^O_2_ and O_2_•^−^, respectively, in the four remediation systems. From the peak intensity of the EPR spectra, the signals of O_2_^−^· and ^1^O_2_ for both CS and CSB were weak, indicating a low efficiency of UV excitation. After PPy modification, the yields of O_2_^−^· and ^1^O_2_ for PPy-CS were increased by 3.2 times and 2.8 times, respectively, while those for PPy-CSB were increased by 2.1 times and 1.4 times, respectively. As a result, the total yield of reactive oxygen species (ROS) for PPy-CS and PPy-CSB was basically equivalent. Specifically, the biomass-based system was dominated by the electron transfer pathway for O_2_^−^· generation, whereas the biochar-based system was dominated by the energy transfer pathway for ^1^O_2_ generation. It should be noted that in the biochar system, ^1^O_2_ did not directly participate in Cr(VI) reduction; the electrons for Cr(VI) reduction were primarily derived from PFR-mediated direct electron transfer and the oxidation of phenolic hydroxyl groups by Cr(VI), with O_2_^−^· serving as an auxiliary reductant. Although the two systems differed in ROS generation pathways, their total available electron capacities for Cr(VI) reduction were ultimately comparable.

### 3.4. FTIR Analysis

**Figure 7:**
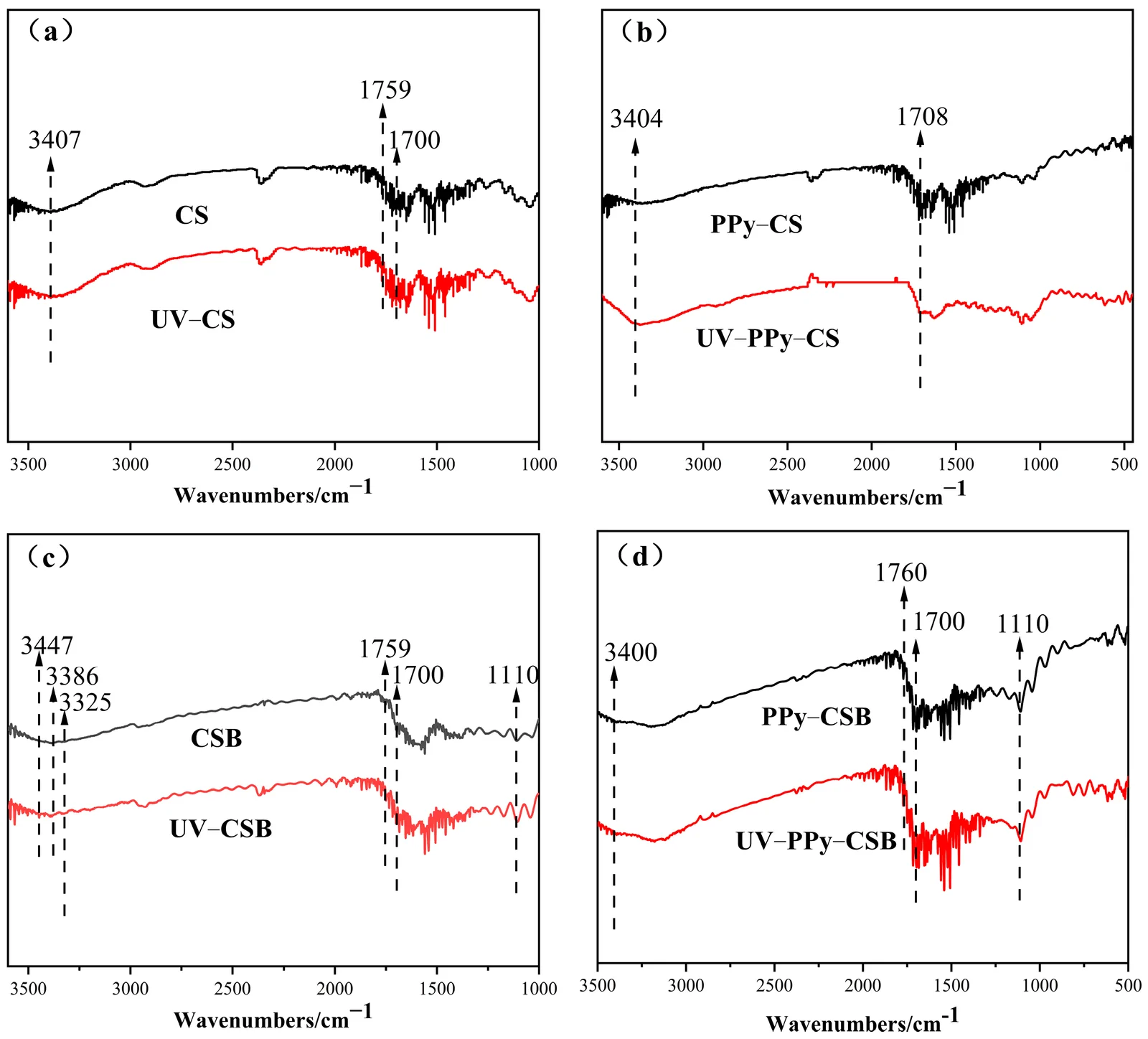
Infrared spectra of remediation materials before and after ultraviolet irradiation. (**a**) CS; (**b**) PPY-CS; (**c**) CSB; (**d**) PPY-CSB.

Figure 7 shows the FTIR spectra of CSB, PPy-CSB, CS, and PPy-CS before and after UV irradiation. The results indicate that UV-CSB exhibited more intense absorption peaks than CSB at 3447, 3386, and 3325 cm^−1^ (-OH stretching), 1700–1759 cm^−1^ (C=O stretching of carboxyl), and 1110 cm^−1^ (C-O vibration) [34]. The peak intensities of UV-PPy-CSB at 3400 cm^−1^ (-OH vibration), 1700~1760 cm^−1^ (C=O vibration), and 1110 cm^−1^ (C-O vibration) were stronger than those of PPy-CSB. In addition, the peak intensities of UV-CS at 3407 cm^−1^ (-OH vibration) and 1700~1759 cm^−1^ (C-O vibration) were higher than those of CS. The peak intensities of UV-PPy-CS at 3404 cm^−1^ (-OH vibration) and 1708 cm^−1^ (C-O vibration) were higher than those of PPy-CS. In summary, the peaks associated with -OH, C=O, and C-O groups on the surface of the adsorbent were more intense after UV irradiation than before. This indicated that UV irradiation enhanced the oxygen-containing functional groups to some extent, thereby promoting the reduction or adsorption of Cr(VI) in soil.

### 3.5. Reaction Mechanism Speculation

For biomass-based materials (CS/PPy-CS), the CS contained only a small number of isolated natural photosensitive groups (benzene rings and carbonyl groups), leading to a weak reduction capacity [35]. After modification with PPy, a stable conjugated long-chain structure was formed on the material surface, which could be efficiently excited to generate abundant carbon-centered free radicals under UV irradiation [36]. These radicals transferred electrons to dissolved oxygen in the soil through the electron transfer pathway, preferentially producing O_2_^−^·, accompanied by a small amount of ^1^O_2_. The strongly reductive O_2_^−^· could directly reduce Cr(VI) to low-toxic and stable Cr(III). Furthermore, ^1^O_2_ can also oxidize the C=C double bonds and heteroatom-containing groups on the material surface; the resulting oxidation intermediates further decompose into oxygen-containing functional groups such as hydroxyl and carboxyl groups, which further immobilized Cr(III) on the material surface via electrostatic adsorption and complexation. The possible reaction mechanism process of biomass-based materials is shown as follows.

(3) $$CS/PPy-CS\overset{UV}{\to }PFR\cdot$$

(4) PFR⋅+O23→O2−⋅

(5) $$O_{2}^{-}\cdot +Cr\left(VI\right)\to Cr\left(III\right)$$

(6) O21→−OH/−COOH→ComplexationImmobilized CrIII

Regarding the biochar-based materials (CSB/PPy-CSB), the CSB possessed a conjugated carbon skeleton and surface oxygen-containing functional groups formed during high-temperature pyrolysis [37]. After PPy modification, a π-π conjugation interaction was established between PPy and the biochar framework, which effectively suppressed the recombination of photogenerated electron–hole pairs and significantly boosted the generation and stabilization efficiency of free radicals [38]. Under UV irradiation, this system accomplishes the reduction and immobilization of Cr(VI) through multiple synergistic pathways. Initially, UV excitation of the conjugated carbon skeleton and PPy photosensitive moieties generates carbon-centered and oxygen-centered PFRs, which act as direct electron donors to reduce Cr(VI) to Cr(III). Meanwhile, the abundant surface C–OH groups on biochar serve as electron donors; they are directly oxidized to C=O groups by the highly oxidative Cr(VI), and the electrons released in this process concurrently drive the reduction of Cr(VI). XPS analysis further verified this transformation, as evidenced by a dramatic decline in the relative proportion of C–OH from 71.96% to 26.65% and a concomitant rise in C=O from 8.02% to 46.26%. Furthermore, PPy functions as a photosensitizer. Upon UV excitation, it converts ground-state triplet oxygen (^3^O_2_) to singlet oxygen (^1^O_2_) via energy transfer. As an electrophilic oxidant, ^1^O_2_ reacts with the C=C double bonds in the biochar carbon skeleton and the N-containing heteroatomic moieties in PPy, forming peroxide intermediates that further decompose into oxygen-containing functional groups such as C=O and COOH groups. These functional groups stably immobilize the generated Cr(III) through complexation and electrostatic adsorption, thereby driving the reduction reaction forward and achieving efficient synergistic reduction–stabilization removal of Cr(VI). The possible reaction mechanism process of the biochar-based materials is shown as follows.

(7) $$CSB/PPy-CSB\;\overset{UV}{\to }PFR\cdot +e^{-}$$

(8) $$C-OH\to C=O+e^{-}$$

(9) $$Cr\left(VI\right)+e^{-}\;\to Cr\left(III\right)$$

(10) PPY+O23 →UVO21

(11) O21+C=C →C=O+COOH

(12) $$Oxygen-containing\;groups\;\overset{Adsorption}{\to }\;Immobilized\;Cr\left(III\right)$$

In summary, the two systems differ fundamentally in their Cr(VI) remediation mechanisms. Biomass-based materials (CS/PPy-CS) follow an electron-transfer-dominated pathway in which UV-generated photoelectrons preferentially reduce dissolved oxygen to O_2_^−^·, the primary reductant of Cr(VI). Biochar-based materials (CSB/PPy-CSB), however, are driven mainly by PFR-mediated direct electron transfer and electron release from the Cr(VI)-induced oxidation of phenolic hydroxyl groups; their higher ^1^O_2_ yield contributes solely to Cr(III) immobilization via carbon skeleton oxidation and functional group generation, rather than direct Cr(VI) reduction. Nevertheless, both systems deliver comparable total electron availability and effective Cr(VI) reduction and immobilization.

### 3.6. Comparisons

Table 3 presents a summary of the treatment effects of different adsorbents on Cr(VI) under various conditions. The table shows that different light conditions greatly impacted the treatment effect of Cr(VI). For example, a higher power and shorter wavelength of the UV lamp generated more energy, which could improve the treatment effect of Cr(VI). However, excessive UV lamp power might raise the reaction temperature, potentially damaging the structure and functionality of remediation materials. This could lead to higher energy consumption and costs, resulting in the inefficient use of resources. Furthermore, by comparing with other studies, it was found that the majority of the literature on enhancing the treatment efficacy of Cr(VI) by UV light concentrated on Cr(VI) contamination in aqueous environments. In contrast, this study evaluated the effects of combining UV radiation with remediation materials on Cr(VI) contamination in soil and demonstrated that UV radiation effectively improved soil remediation results.

## 4. Conclusions

The research demonstrated that UV irradiation could effectively improve the remediation performance for the treatment of Cr(VI)-contaminated soil. Under the remediation conditions of CSB, PPy-CSB, CS, and PPy-CS, the application of UV irradiation resulted in an increase of 18.7%, 7.6%, 11.0%, and 8.2% in the treatment effects of Cr(VI) in soil, respectively. EPR analysis indicated that the presence of both ^1^O_2_ and $O_{2}^{-}$· were detected in the material system following UV irradiation. Although the active oxygen pathways produced by biomass-based material and biochar-based material systems were different, the total amount was similar. XPS analysis revealed that without remediation agents, UV irradiation promoted the conversion of Cr(III) to Cr(VI) in soil. However, under the PPy-CSB and PPy-CS remediation systems, UV irradiation could promote the conversion of Cr(VI) to Cr(III). Additionally, UV-PPy-CSB and UV-PPy-CS exhibited different oxygen reaction pathways due to variations in the distribution of carbon functional groups such as C-OH and C=O. The UV irradiation could activate the reactions between Cr(VI) and remediation agents in the soil, and the UV-CS achieved a Cr(VI) removal performance comparable to that of the PPy-CSB. UV irradiation offers an efficient and promising technological approach for the remediation of Cr(VI)-contaminated soil.

## Figures and Tables

**Table 1:** Basic properties of the background soil.

Basic Indicators	Content
pH	7.8 ± 0.08
Alkaline nitrogen (mg·kg^−1^)	17.85 ± 0.71
Organic matter (g·kg^−1^)	10.23 ± 0.12
Soil capacity (g·cm^−3^)	1.3 ± 0.09
Water content (%)	2.13 ± 3.20
Total Pb (mg·kg^−1^)	3.87 ± 0.24
Total Cu (mg·kg^−1^)	8.67 ± 0.53
Total Cr (mg·kg^−1^)	34.11 ± 2.36
Cr(VI) (mg·kg^−1^)	-

**Table 2:** Basic properties of the simulated contaminated soil.

Basic Indicators	Cr(VI) Concentrations
Testing total Cr(VI) concentrations (mg·kg^−1^)	582.36 ± 4.12
pH	8.13 ± 0.04
Alkaline nitrogen(mg·kg^−1^)	17.56 ± 0.56
TCLP-Cr(VI) (mg·kg^−1^)	437.71 ± 2.26
Total Cr(VI) concentrations (mg·kg^−1^) after one week	512.73 ± 3.06

**Table 3:** Comparison of the adsorption capacity of chromium by UV radiation.

Adsorbents	Light Conditions	Pollution Type	Q_m_ (mg·g^−1^)	Reference
Sludge biochar	Deuterium lamp with a power of 4 W, a wavelength of 190–400 nm, an irradiation time of 6 h, and an irradiation distance of 15 cm.	Water pollution	1.97	[39]
Corn straw biochar (BC)	Used a 250 W UV lamp with a wavelength of 365 nm, an irradiation time of 24 h, and an irradiation distance of 40 mm.	Water pollution	20.04	[40]
Titanium metal organic frameworks (MIL-125(Ti) + TA)	Used a UV lamp with a power of 64 W, a wavelength of 420 nm, and an irradiation time of 2 h.	Water pollution	9.94	[41]
TiO_2_/CoFe_2_O_4_/Ag nanocomposites	Used UV lamps with a power of 120 W and a wavelength of 350–390 nm.	Water pollution	47.55	[42]
PPy-CS	Used a power of 30 W, an ultraviolet lamp with a wavelength of 254 nm, an irradiation time of 2 h, and an irradiation distance of 30 cm.	Soil pollution	7.55	This Work
PPy-CSB	Used a power 30 W, an ultraviolet lamp with a wavelength of 254 nm, an irradiation time of 2 h, and an irradiation distance of 30 cm.	Soil pollution	7.48	This Work

Note: The UV irradiation conditions listed in this table are presented as reported in the respective references. Quantitative comparison of UV irradiation energy is not directly feasible, owing to the absence of essential experimental parameters in most of the referenced studies.

## Data Availability

The original contributions presented in this study are included in the article materials. Further inquiries can be directed to the corresponding author.
